# Supplementary material for: Multimodal prehabilitation in people awaiting acute inpatient cardiac surgery: Study protocol for a pilot feasibility trial (PreP-ACe)
Source: PLoS One. 2025 Mar 10;20(3):e0307341. doi: 10.1371/journal.pone.0307341 (PMC11892877; doi:10.1371/journal.pone.0307341)
Supplement: S1 Table — (PDF) [file pone.0307341.s002.pdf]

Table S1: Outcome measures and time point assessments for individuals recruited to PreP-ACe

Variable/ Timepoint	Baseline	Day 7 of intervention	Day 14 of intervention/ day before surgery	End of intervention
**Physical/ exercise**				
6MWT	X	X	X	
Hand grip	X	X	X	
Spirometry	X	X	X	
**Clinical psychology**				
CAQ	X		X	
EQ-5D-5L (QoL)	X		X	
**Clinical Outcomes**				
Length of hospital stay				X
Adverse events	X	X	X	X
Post-operative complications				X
Re-admission				X

6MWT: 6 minute walk test [7, 8, 36] Hand Grip Test [38, 42] Spirometry [37] CAQ: Cardiac Anxiety Questionnaire [40, 41] EQ-5D-5L: Health and quality of life questionnaire [39]
