# Supplementary material for: Multimodal prehabilitation in people awaiting acute inpatient cardiac surgery: Study protocol for a pilot feasibility trial (PreP-ACe)
Source: PLoS One. 2025 Mar 10;20(3):e0307341. doi: 10.1371/journal.pone.0307341 (PMC11892877; doi:10.1371/journal.pone.0307341)
Supplement: S2 Table — (PDF) [file pone.0307341.s003.pdf]

Table S2: Stop-Go Criteria

Go: Continue recruitment; **Modify**: Review the trial protocol / processes. Changes will be reviewed by the trial management group; **Stop**: The trial will close if the trial management group demonstrate the intervention is not safe or feasible.

Outcome criteria	Go	Modify	Stop
**Median adherence to intervention (%)**	≥70%	50-69%	<50%
**Median tolerability to intervention (%)**	≥70%	50-69%	<50%
**Serious adverse events attributable to intervention**	0-1	2-3	>3

✓Median adherence to programme: defined as number of sessions the participant attends divided by the total number of sessions offered;

✓ Median tolerability to programme: defined as the number of minutes the participant exercises for divided by the total number of minutes in the whole programme. We will also record weekly mean changes in HR, power output, and RPE over the intervention;

✓Serious adverse events: defined as untoward medical occurrences related to the intervention either during or 30 minutes after intervention which leads to significant morbidity or mortality. This includes major adverse cardiovascular events and escalation of urgency of surgery.
