# Supplementary material for: Multimodal prehabilitation in people awaiting acute inpatient cardiac surgery: Study protocol for a pilot feasibility trial (PreP-ACe)
Source: PLoS One. 2025 Mar 10;20(3):e0307341. doi: 10.1371/journal.pone.0307341 (PMC11892877; doi:10.1371/journal.pone.0307341)
Supplement: S3 File — (PDF) [file pone.0307341.s006.pdf]

Version number and date: 06/11/2023 (2)

[IRAS ID: 330859]

CONSENT FORM

Title of study: PreP-ACe

Name of Researcher(s): Dr Sarah Raut, Prof Lee Ingle, Prof Gerard Danjoux, Dr Simon Davies, Dr David Yates

Please initial box

1. I confirm that I have read the information sheet dated 06/11/2023 version 4 for the above study. I have had the opportunity to consider the information, ask questions and have had any questions answered satisfactorily. ☐

2. I understand that my participation is voluntary and that I am free to withdraw at any time without giving any reason, without my medical care or legal rights being affected. I understand that the data I have provided up to the point of withdrawal will be retained. ☐

3. I understand that relevant sections of my medical notes and data collected during the study, may be accessed by individuals from regulatory authorities or from the NHS Trust, where it is relevant to my taking part in this research. I give permission for these individuals to have access to my records. ☐

4. I understand that the research data, which will be anonymised (not linked to me), will be retained by the researchers and publicly disseminated. ☐

5. I understand that my data from this research may be shared with others to support other research in the future. (optional) ☐

6. I understand that my personal data will be kept securely in accordance with data protection guidelines, and will only be available to the immediate research team ☐

7. I give permission for the collection and use of my data to answer the research question in this study. ☐

8. I agree to take part in the above study.

____________________	____________________	____________________
Name of Participant	Date	Signature

____________________	____________________	____________________
Name of Person taking consent	Date	Signature

9. I agree for my data to be shared with other researchers to support future research.

____________________ ____________________ ____________________

Name of Participant	Date	Signature
Name of Person taking consent	Date	Signature

10. I agree to participate in the feedback of the program.

Name of Participant	Date	Signature
Name of Person taking consent	Date	Signature
