# Supplementary material for: Multimodal prehabilitation in people awaiting acute inpatient cardiac surgery: Study protocol for a pilot feasibility trial (PreP-ACe)
Source: PLoS One. 2025 Mar 10;20(3):e0307341. doi: 10.1371/journal.pone.0307341 (PMC11892877; doi:10.1371/journal.pone.0307341)
Supplement: Attachment — Submitted filename: Response to reviewer 3.docx [file pone.0307341.s010.docx]

Dear Reviewer,

Thank you very much for your time and feedback on my protocol- Multimodal prehabilitation in People awaiting acute inpatient cardiac surgery: study protocol for a pilot feasibility trial (PreP-ACe). I have taken your comments into consideration and amended where appropriate. Please see below the responses to your feedback.

Reviewer #3: Well thought out and good idea to consider prehabilitation in selected individuals waiting for cardiac surgery. I greatly appreciate the authors addressing previous reviewer comments. I would recommend to include the demographics of the patient population - age, sex, BMI; some of the common medical comorbidities that are associated with cardiac surgery - HTN, DM2, PAD, AKI or CKD, HLD, COPD, or on O2 at baseline; albumin level; LV function prior to surgery, presence of any IV or subcutaneous antiplatelets agents or anticoagulants, risk of falls/fragility, are they functionally independent prior to this admission. If they don't have these medical comorbidities, these patients would tend to participate more in the prehabilitation program and you would only have stable patients who would do well in the long run regardless if they undergo prehabilitation or not. It would be good to see these data in the feasibility study.

Thank you very much for your feedback. We will consider all acute patients waiting for inpatient cardiac surgery. When we report the outcome of the feasibility study, we will include age, sex, BMI, co-morbidities etc as stated above. We do expect patients who participate to have a degree of stability (eg: asymptomatic at rest, sepsis with ongoing treatment).

It is uncommon to wait beyond 7 days for an urgent cardiac surgery in US or some Asian countries.

This study is conducted in the UK where the main healthcare provider is publicly funded. Unfortunately, waiting times are a feature of public funded healthcare.

"Only 34% of acute patients receive their surgery within 7 days in 2019/2020." Things are different post COVID - you can consider what's your current % of these patients getting their surgery within 7 days. If this % way higher than before, you may have difficulty recruiting patients beyond the feasibility trial.

Thank you very much for your feedback. We did consider this. As such we have done some scoping work. We analysed patients who came to our hospital in 2022 and tried to describe them based on their presentation, co-morbidities, type of surgery and outcome. As this is a separate piece of work currently under review, we are unable to make reference to this in the protocol.
