# Supplementary material for: Multimodal prehabilitation in people awaiting acute inpatient cardiac surgery: Study protocol for a pilot feasibility trial (PreP-ACe)
Source: PLoS One. 2025 Mar 10;20(3):e0307341. doi: 10.1371/journal.pone.0307341 (PMC11892877; doi:10.1371/journal.pone.0307341)
Supplement: Attachment — Submitted filename: Prep-Ace reviewer feedback.docx [file pone.0307341.s009.docx]

Dear Reviewers,

Thank you very much for your time and feedback on my protocol- Multimodal prehabilitation in People awaiting acute inpatient cardiac surgery: study protocol for a pilot feasibility trial (PreP-ACe). I have taken your comments into consideration and amended where appropriate. Please see below the responses to your feedback.

Reviewer #1: Thank you for the invitation to review this interesting study protocol. The rationale is clearly articulated that this is a novel intervention and important area of research. I have made a few comments below for your consideration.

Sample size - I understand approximately 100 patients were admitted to CHH per year and would be available for recruitment but what proportion of these would be likely to be eligible e.g. how many had a condition unlikely to be treated with surgery? How many underwent surgery in less than 5 days post-admission?

We are currently looking to publish data from our scoping work as such I am unable to provide too many detail. Approximately 100 patients are admitted directly to CHH for acute cardiac surgery in a year. 22% underwent surgery within the week (as per NICOR), of the remaining patients, some would have been too unwell to participate in the program or require medical optimisation that would impinge on their ability to participate in the program.

PPIE - As written it in unclear how feedback from PPIE members influenced the study or intervention design - for example did PPIE members feed into the content of the exercise programme? It may also be worth commenting on how representative PPIE members were of the population e.g. considering sex, race, economic status. Will PPIE members have a role throughout the trial?

This is now included in the PPIE section. With a breakdown of the patient ages and type of surgery. We did not collect data on economic status. Due to PPIE and staff feedback, we have removed the nutritional element of the study. This has been explained in the intervention section (nutrition). There may be an opportunity for education classes in the future but this is not feasible with our current trial. Our psychoeducation booklet is designed to provide information on their admission as well as meditation.

Data collection - Would patients usually be referred to cardiac rehab on discharge from inpatient care? - if so it would be interesting to collect data on uptake. It is possible that those who receive prehab refuse cardiac rehab (this was observed by Greening et al 2018 with regards to pulmonary rehab), this would be an unintended consequence.

Currently, all patients would be referred to cardiac rehab post discharge. This would be an interesting point and we can certainly try to follow these patients up post discharge.

Data collection - the content of the intervention and the outcome measures do not appear to be completely aligned - The exercise intervention includes balance and strength training yet there is no outcome measures to assess strength (although grip strength could be a proxy for lower-limb strength) or balance (e.g. TUG, SLS). Did the authors considered assessing MIP as IMT is part of the intervention?

When designing the study, we did initially consider TUG. However, after much consideration, this was removed due to concerns of postural hypotension in this population. Again, with SLS, we were not sure about the safety of performing this test in acute inpatients. It maybe that this will be added in the future study as we gain more insight and confidence.

Yes, we did consider MIP but the equipment was not readily available. Due to the restricted budget and available personel, we had to rationalise our interventions and outcome measures. If we gain future funding, this could change.

Intervention - please consider using thee TiDiER checklist. Currently there is no description of the balance/flexibility exercises to be performed.

TiDiER checklist included. The balance and flexibility exercise will be based on the physiotherapist assessment.

In a feasibility trial I would expect to see "stop go criteria" or progression criteria?

Agreed. We have added criteria as you indicate.

End of study and Statistical analysis - this is the first time a qualitative component is mentioned? In the study design it should be clear that a mixed-methods approach is being applied. Who is conducting the interviews? Where? What is the purpose of the interviews? What analysis will be applied?

The method section of the abstract does mention seeking feedback from the participants. On figure 1: SPIRIT schedule, there is a section on Post study feedback at the end of the intervention. The authors accept this could be made clearer; as such, a section is added under study procedures and study outcomes. The researcher SR will be seeking feedback from the participants at then end of the intervention to ascertain practicality, acceptability and enjoyment of the program as well as feedback on the psychoeducation booklet. Whenever possible, data will be provided as percentages or counts. However, free-prose responses will be transcribed verbatim.

Reviewer #2: As a pilot feasibility study, no formal sample size calculation is required. However, since the primary objective of this study is to determine the feasibility of a prehabilitation programme, it is expected to see how the feasibility will be quantified. For example, how big of the enrollment rate or completion rate would establish the feasibility. Even though the statistical analysis for such a pilot study is mainly descriptive, it would be better to have more details such as all 95% confidence intervals will be reported for any rates. There would be longitudinal data (such as QoL) collected over time and hence proper models for such data should be used in order to better estimate the change in QoL after the programme. Consultation with and/or the involvement of a professional biostatistician in the study team is strongly recommended.

Thank you. We will look to include a biostatistician as the project progresses and we determine whether we wish to pursue a full RCT. In relation to your example, we are currently not collecting long-term QoL data. The end of study will be the day before the surgery. It would be interesting for future study to perform a case- control study to assess QoL change after the program.
